# Navigating the Boundaries of Teleconsultation—Capabilities, Limitations, and Pathways for Improvement: Qualitative Study of the Experiences of Patients With Stroke

**DOI:** 10.2196/75841

**Published:** 2025-09-18

**Authors:** Arkers Kwan Ching Wong, Xiaomin Lu, Joyce Xiaoli Wang, Rose Sin Yi Lin, Jing Jing Su, Robbie Mian Wang, Vivian Wai Yan Kwok

**Affiliations:** 1 Hong Kong Polytechnic University Hung Hom China (Hong Kong); 2 University of Rochester Medical Center New York United States; 3 Tung Wah College Homantin China (Hong Kong); 4 Queen Elizabeth Hospital Homantin China (Hong Kong)

**Keywords:** teleconsultation, stroke rehabilitation, patient experience, qualitative research, digital health

## Abstract

**Background:**

Survivors of stroke often face persistent challenges accessing postdischarge care due to mobility limitations, transportation burdens, and inflexible scheduling. Teleconsultation has emerged as a potential solution to improve continuity of care, but its perceived strengths and limitations from the patient perspective remain insufficiently understood.

**Objective:**

This study aimed to explore the experiences of survivors of stroke with a nurse-led teleconsultation program to (1) identify perceived capabilities; (2) understand limitations in usability, accessibility, and clinical function; and (3) generate patient-informed recommendations for improvement.

**Methods:**

A qualitative study was embedded within a 3-month nurse-led teleconsultation intervention delivered by advanced practice nurses. A total of 21 survivors of ischemic stroke (aged 45-76 y; female: n=11, 52%) who had preserved cognitive function (Montreal Cognitive Assessment score ≥22) and smartphone access participated in 6 focus groups conducted via Zoom. Data were analyzed thematically using an established framework. Data saturation was achieved.

**Results:**

Participants widely valued teleconsultation for reducing logistical burdens; enhancing access; and offering a more comfortable, emotionally supportive setting for follow-up care. Many reported increased awareness and motivation for self-monitoring. However, limitations included an inability to perform physical assessments or respond to emergencies; digital and usability barriers, especially among older users; and scheduling inflexibility. Participants emphasized the need for patient-initiated follow-up mechanisms, physician collaboration for medication management, and greater support for users considered digitally marginalized. They also highlighted the potential of teleconsultation to serve as a triage tool, reserving in-person care for complex cases.

**Conclusions:**

Nurse-led teleconsultation was perceived as a convenient and supportive modality for poststroke care, particularly for stable follow-ups and psychosocial support. However, its long-term viability depends on addressing clinical and technical limitations, enhancing user autonomy, and integrating interdisciplinary input. By centering the lived experiences of survivors of stroke, this study offers concrete recommendations to guide the development of more inclusive, responsive, and patient-centered teleconsultation models.

## Introduction

### Background

Stroke, a cerebrovascular event characterized by sudden neurological deficit, persists as a leading contributor to global mortality and disability, with an estimated 12.2 million incident cases and 6.6 million deaths annually worldwide [1]. After acute care and early rehabilitation, survivors of stroke enter a chronic phase of recovery characterized by diverse challenges, including motor impairment, aphasia, cognitive decline, and psychological distress, which collectively diminish functional independence and quality of life [2-4]. Particularly concerning is the high recurrence rate of stroke, affecting up to 51.3% of survivors, which further exacerbates disability and diminishes quality of life [5].

Nurse-led clinics have emerged as a key model for delivering poststroke follow-up care, providing services that involve comprehensive screening for complications, diligent management of risk factors such as hypertension and hypercholesterolemia, lifestyle modifications, medication adherence education, and psychosocial support [5,6]. Evidence shows that these services improve the functional and psychosocial outcomes for survivors of stroke and reduce readmission rates [5,7,8]. However, the face-to-face delivery mode encounters substantial barriers, including patient mobility limitations, transportation challenges, and prolonged waiting times at health care facilities, all of which hinder patients with stroke from attending their appointments on time [9,10]. In response, the health care sector has increasingly adopted teleconsultation as a flexible and cost-effective alternative to continue providing care to patients with stroke.

Teleconsultation is defined as a medical consultation conducted remotely between a health care professional and a patient using communication technology [11]. This innovative approach not only enhances accessibility and continuity of care for survivors of stroke but also offers substantial benefits, including increased convenience; broader access to specialized care, which is especially critical for patients in rural or underserved areas; and significantly reduced health care costs [12]. Moreover, the flexibility of telecare schedules can accommodate patients’ needs better, thus minimizing the impact of common barriers such as transportation and mobility limitations [13]. Overall, the adoption of telecare has the potential to transform poststroke care, making it more responsive and accessible for those in need.

While the convenience and cost-effectiveness of teleconsultation have been widely recognized, many believe that it cannot fully replace in-person care because of clinical applicability constraints; for instance, its reliance on digital platforms creates accessibility barriers among older adults and populations with limited technological literacy [14]. Due to technological constraints such as image quality degradation and connectivity disruptions, telehealth systems cannot fully replicate all components of physical examinations [15,16]. The effective implementation of teleconsultation requires careful consideration of both technological capabilities and limitations. Previous studies have explored facilitators and barriers to telecare, including technological limitations, medication adherence challenges, and patient engagement issues [17-19]. However, there remains a critical knowledge gap in understanding how these limitations are experienced by patients with stroke themselves in the context of nurse-led teleconsultation services. Although prior research has examined barriers to telecare broadly, few studies have focused on the lived experiences of survivors of stroke participating in structured nurse-led teleconsultation programs. This lack of patient-centered qualitative evidence limits our understanding of how teleconsultation works in real-world rehabilitation contexts. The patient perspective is particularly valuable in identifying practical challenges and opportunities for service improvement because it provides direct insight into how technical and communicative aspects of teleconsultation affect the quality and effectiveness of care received [20].

### Objectives

To better address the issues related to the advantages and limitations of teleconsultation and explore pathways for improvement, we conducted a qualitative study among survivors of stroke involved in a nurse-led teleconsultation program. We aimed to (1) identify the perceived capabilities of teleconsultation in enhancing poststroke care accessibility and satisfaction, (2) delineate its limitations in addressing complex care needs, and (3) uncover actionable pathways for improvement based on patients’ perspectives. This study was guided by the following research questions:

1. What are the perceived capabilities of nurse-led teleconsultation in supporting poststroke care?
2. What limitations are identified by users?
3. What suggestions do survivors of stroke offer for improving teleconsultation services?

The findings gained from this study will inform policy decisions, refine telehealth protocols, and advance equitable stroke rehabilitation strategies tailored to diverse patient populations.

## Methods

### Overview

This study used a qualitative descriptive approach embedded within a larger randomized controlled trial evaluating a nurse-led teleconsultation intervention [21]. The qualitative arm aimed to elicit in-depth perspectives on user experience, including strengths and areas for improvement.

### Participants and Recruitment

Participants were recruited from a regional public hospital’s stroke unit in Hong Kong, as part of a larger randomized controlled trial. Specifically, individuals who had completed the nurse-led teleconsultation program and met the eligibility criteria were purposively selected to join the focus groups.

Eligibility criteria included the following: (1) confirmed ischemic stroke diagnosis within the past month, (2) cognitive competency (Montreal Cognitive Assessment score ≥22), (3) age ≥18 years, (4) smartphone ownership with internet access, and (5) anticipated discharge within one week. Exclusion criteria were significant sensory deficits and bedbound status. Stroke nurses introduced the study to eligible patients, and a research assistant conducted informed consent procedures.

As part of the clinical study screening process, basic demographic data were collected at the time of recruitment by trained research staff using a standardized intake form. This included age, sex, stroke type and history, and educational level.

### Intervention Description

The intervention consisted of 3 monthly nurse-led video consultations delivered over a 3-month period through a secure, encrypted telecare platform. Each session lasted approximately 60 minutes and included education on secondary stroke prevention, review of laboratory results, medication adherence counseling, and discussion of individualized risk factor management. Family members or caregivers were encouraged to attend. Nurses referred participants to relevant services if clinical deterioration was observed or if complex needs were identified.

All consultations were conducted by the same group of advanced practice nurses, each with more than 20 years of clinical experience in stroke care. While patients were generally seen by the same nurse across sessions when possible, some were followed by different team members due to scheduling needs. All nurses had received structured training in telehealth delivery, including use of the digital platform and strategies for effective remote communication and patient education.

### Data Collection

Six semistructured focus groups were conducted via Zoom (Zoom Video Communications, Inc) between November and December 2024. Each group comprised 2 to 5 participants, with sessions lasting 60 to 90 minutes. Discussions were guided by a semistructured interview protocol (Multimedia Appendix 1) developed by the research team. Key domains included patient perceptions of teleconsultation usability, barriers and facilitators, areas of satisfaction and concern, and recommendations for improvement. All sessions were audio recorded, transcribed verbatim, and translated from Cantonese to English by two bilingual researchers fluent in both English and Cantonese. Back translation was performed to ensure fidelity.

### Data Analysis

Verbatim transcripts were analyzed using the 6-phase thematic analysis framework developed by Braun and Clarke [22]. Two independent coders (XL and JXW) performed initial coding in NVivo 12 (Lumivero). Codes were reviewed and refined into overarching themes through iterative team discussions. Discrepancies were resolved through consensus or arbitration by a third researcher (AKCW).

Data saturation was reached by the sixth focus group, as no new codes or themes emerged during analysis of the final transcripts. Reflexivity was maintained through ongoing team discussions to reflect on researchers’ assumptions and minimize bias. Credibility was enhanced through analyst triangulation, and confirmability was supported by verbatim use of participant quotes. Dependability was ensured via a documented coding process, and transferability was supported by providing detailed participant characteristics and study context.

### Ethical Considerations

Ethics approval was granted by the ethics committee of the Hong Kong Polytechnic University (HSEARS20211207003). All participants provided written informed consent after receiving information on study aims, procedures, risks, and confidentiality. Data were deidentified and securely stored. Each participant received a supermarket voucher worth HKD $50 (US $6.42) as compensation. No identifying images or personally revealing information were included in the manuscript or supplementary materials; all quotations were anonymized, and pseudonyms were used throughout.

## Results

### Participant Characteristics

A total of 21 participants (aged 45-76 y; female: n=11, 52%) took part in the study. All participants had experienced ischemic strokes, with 71% (15/21) being first-time survivors of stroke. Their educational background varied: of the 21 participants, 3 (14%) had completed primary school, 13 (62%) had secondary education, and 5 (24%) held tertiary-level qualifications. Participants were grouped into 6 focus groups of 2 to 5 individuals. All participants completed the 3-month teleconsultation program. Table 1 provides their demographic characteristics.

**Table 1:** Demographic characteristics of survivors of stroke participating in nurse-led teleconsultation focus groups (Hong Kong, November-December 2024).

Patient ID	Focus group ID	Age (years)	Sex	First-ever stroke	Stroke type	Educational level
P01	1	69	Female	No	Ischemic	Secondary
P02	1	70	Male	Yes	Ischemic	Tertiary or above
P03	1	60	Male	Yes	Ischemic	Secondary
P04	1	70	Female	Yes	Ischemic	Secondary
P05	1	58	Female	Yes	Ischemic	Secondary
P06	2	69	Male	No	Ischemic	Tertiary or above
P07	2	67	Female	Yes	Ischemic	Primary
P08	2	66	Female	No	Ischemic	Secondary
P09	3	66	Female	No	Ischemic	Secondary
P10	3	62	Male	No	Ischemic	Tertiary or above
P11	3	49	Female	Yes	Ischemic	Secondary
P12	3	61	Male	Yes	Ischemic	Secondary
P13	3	45	Female	Yes	Ischemic	Secondary
P14	4	64	Female	Yes	Ischemic	Primary
P15	4	73	Male	Yes	Ischemic	Secondary
P16	4	76	Male	Yes	Ischemic	Secondary
P17	5	57	Female	Yes	Ischemic	Tertiary or above
P18	5	62	Male	Yes	Ischemic	Primary
P19	5	51	Male	No	Ischemic	Secondary
P20	6	66	Male	Yes	Ischemic	Tertiary or above
P21	6	63	Female	Yes	Ischemic	Secondary

### Research Question 1: Capabilities of Nurse-Led Teleconsultation

#### Theme 1: Enhancing Accessibility and Convenience

Participants consistently reported that teleconsultation significantly improved health care accessibility by eliminating the need for physical travel. This was especially meaningful for those with mobility impairments or those living far from health care facilities. Several individuals noted that the reduced burden of transportation allowed for more consistent engagement with poststroke care:

Stroke patients often face mobility challenges, as their limbs may not be as agile, and some may still require the use of a cane. Traveling long distances to the hospital can be difficult for them.

P07, focus group 2; female, aged 67 y; primary education

The convenience of home-based care also helped avoid long hospital wait times and offered scheduling flexibility. For working participants, being able to consult from their workplace reduced disruption to their daily routine:

I was ready in 5 to 10 minutes...everything set up beforehand.

P16, focus group 4; male, aged 76 y; secondary education

I did it straight from my office during a break at work.

P19, focus group 5; male, aged 51 y; secondary education

#### Theme 2: Enhancing Patient Experience and Satisfaction

Teleconsultation fostered a more relaxed and psychologically safe environment, which contributed to more satisfying and open interactions with health care professionals. Patients appreciated the continuity of care and emotional support provided by nurses:

I feel more relaxed during teleconsultation, maybe because I’m at home. The nurse was very caring and followed up closely, which helped relieve my stress.

P15, focus group 4; male, aged 73 y; secondary education

The setting reduced consultation-related anxiety, which participants frequently associated with traditional hospital visits:

Going to the hospital for a consultation is highly stressful, but video consultations eliminate this psychological burden.

P18, focus group 5; male, aged 62 y; primary education

#### Theme 3: Supporting Self-Management and Health Literacy

Participants shared that the teleconsultation model empowered them to take greater responsibility for their health. Nurses’ personalized questions and explanations increased their awareness of key health behaviors, including blood pressure monitoring, weight control, and dietary choices:

When I’m at home on a video call, the nurse carefully asks me about what I have been eating.... I became more aware I should monitor my blood pressure regularly.

P12, focus group 3; male, aged 61 y; secondary education

Information delivered through teleconsultation was often perceived as clearer and more memorable than that received during hospital visits:

The nurse explained in detail the key stroke warning signs and introduced the mnemonic “speech difficulty, face drooping, arm weakness, and time to call 999 [emergency call number in Hong Kong].” This helped me remember the symptoms of a stroke more effectively.

P16, focus group 4; male, aged 76 y; secondary education

#### Theme 4: Efficient Use of Medical Resources

Participants recognized teleconsultation as a resource-efficient approach, particularly for follow-up care and management of stable conditions. By reducing unnecessary hospital visits, teleconsultation contributed to easing system congestion and allowed clinical staff to focus on more critical cases:

Hospitals are extremely crowded nowadays. With teleconsultation, I don’t have to go to the hospital as often, and I can conveniently receive medical advice from home.

P21, focus group 6; female, aged 63 y; secondary education

In addition to personal convenience, participants acknowledged broader system-level benefits, such as shorter waiting times and more efficient hospital workflows.

Overall, these capabilities demonstrate the value of teleconsultation in enhancing convenience, emotional comfort, and self-management. However, as described in the next subsection, participants also encountered significant limitations that impacted their experience and highlighted areas for further improvement.

### Research Question 2: Limitations of Nurse-Led Teleconsultation

#### Theme 1: Clinical Limitations and Emergency Response

Participants acknowledged that while teleconsultation improves accessibility, it is not a substitute for physical clinical assessment. The inability to observe subtle signs or provide immediate intervention was a major concern:

Through teleconsultation, nurses can only see the patient’s face.... But when assessing a stroke patient, being able to observe their movements and body language is crucial.

P01, focus group 1; female, aged 69 y; secondary education

If someone has a stroke or a brain hemorrhage, they need to be treated in a hospital immediately.

P18, focus group 5; male, aged 62 y; primary education

#### Theme 2: Technological Barriers and the Digital Divide

Digital literacy and platform usability emerged as major challenges, particularly for older adults. Participants faced log-in difficulties, connectivity issues, and anxiety about using new technology:

I downloaded the HA Go [the app used in Hong Kong for teleconsultation], but I wasn’t sure how to use it.... We ended up switching to a voice call.

P11, focus group 3; female, aged 49 y; secondary education

#### Theme 3: Scheduling and Caregiver Involvement Constraints

While many participants appreciated the convenience and flexibility offered by teleconsultation, others—particularly those with inflexible work schedules or caregiving responsibilities—found the lack of evening or weekend options to be a barrier to full accessibility:

Yes, it’s all during office hours.... I simply can’t answer calls during the day.

P17, focus group 5; female, aged 57 y; tertiary education

#### Theme 4: Amplified Communication Challenges in Patients With Cognitive or Verbal Impairments

While communication difficulties are common for patients with stroke who have cognitive or speech impairments, participants noted that such challenges can be exacerbated in teleconsultation settings. The inability to observe full-body gestures, subtle facial cues, or receive in-person support can hinder accurate symptom communication and clinical understanding:

Some patients have slurred or distorted speech.... For more severe cases, effective conversation becomes nearly impossible.

P18, focus group 5; male, aged 62 y; primary education

It depends on the patient’s ability to express themselves clearly. If they can’t communicate well and only make vague sounds, you won’t get much information.

P17, focus group 5; female, aged 57 y; tertiary education

Participants acknowledged that while such limitations also exist in face-to-face care, teleconsultation offers fewer adaptive strategies—such as visual scanning, in-person prompting, or the ability to read nonverbal cues—that might help bridge communication gaps.

These limitations—spanning clinical, technological, and communicative challenges—often led to frustration, anxiety, or reliance on caregivers. In the following subsection, we present patient-generated strategies that directly respond to these barriers, suggesting pathways for refining teleconsultation delivery.

### Research Question 3: Pathways for Improvement

#### Theme 1: Improve Technical Support and Accessibility

Participants recommended simplifying app interfaces and offering step-by-step instructions or voice navigation for older users. They also suggested providing devices to bridge the digital divide:

The app should be made simpler and more user-friendly...the process should be as straightforward as possible for elderly patients.

P15, focus group 4; male, aged 73 y; secondary education

#### Theme 2: Flexible Scheduling and Reminders

Participants urged providers to offer consultations during evenings or weekends and send reminders ahead of time:

Are teleconsultations only available during office hours? Can they be extended to the evening?.... A reminder the day before would help.

P17, focus group 5; female, aged 57 y; tertiary education

#### Theme 3: Enhancing Patient Autonomy in Teleconsultation

While participants appreciated the accessibility of nurse-led teleconsultation, many expressed a desire for greater autonomy in managing their care. Specifically, they wished to have more control over initiating consultations rather than relying solely on scheduled appointments determined by health care providers. The current system was seen as reactive and inflexible, with limited opportunity for patients to seek advice when new symptoms or concerns emerged between scheduled follow-ups.

Several participants proposed a more patient-centered model that would allow them to proactively request teleconsultations—through a hotline, mobile app, or online booking system—whenever needed. This autonomy was viewed as essential for timely reassurance, especially among survivors of stroke who may experience anxiety over fluctuating symptoms:

Why not let me take the initiative? If I feel unwell...I can call and check.

P03, focus group 1; male, aged 60 y; secondary education

In addition to initiating follow-ups, participants emphasized the importance of having a choice between teleconsultation and in-person visits, depending on the severity of their condition, personal preferences, and level of comfort with digital tools:

Shouldn’t patients be given the option to choose? If I feel my condition is mild...I should be able to opt for teleconsultation.

P21, focus group 6; female, aged 63 y; secondary education

This theme highlights a gap between the current delivery model and patients’ expectations for flexible, responsive care. Incorporating patient-initiated options and mode-of-care preferences could enhance satisfaction, support self-efficacy, and promote more responsive poststroke care systems.

#### Theme 4: Expanding Interdisciplinary Involvement to Enhance Continuity of Care

While participants valued the support and attentiveness provided by nurses during teleconsultations, many noted limitations in accessing timely prescriptions and specialist input. To improve continuity of care, they expressed a desire for greater involvement from physicians within the teleconsultation system—not as a replacement for nurses but as a complement to their role.

Patients suggested that including physicians in specific scenarios (eg, medication adjustments or complex cases) could streamline decision-making and reduce delays caused by the current referral process. Rather than relying on nurses to relay messages between patients and physicians, participants envisioned a more integrated model where nurses and physicians collaborate in real time:

Nurses still have to consult the doctor, then the doctor replies.... But if I could ask the doctor directly and get an immediate response, it would be completely different.

P18, focus group 5; male, aged 62 y; primary education

In tandem with this, participants advocated for remote prescription services and home delivery of medications, especially for those with mobility limitations:

Doctors could prescribe medication remotely...and it could be delivered to my home.

P04, focus group 1; female, aged 70 y; secondary education

This theme highlights the need for a team-based approach in telehealth that leverages the strengths of both nursing and medical professionals, enabling a more efficient and patient-centered service.

Collectively, these suggestions reflect patients’ lived experiences and offer actionable recommendations to improve inclusivity, flexibility, and interdisciplinary collaboration in teleconsultation.

### Summary of Focus Group Findings

Collectively, these suggestions reflect patients’ lived experiences and offer actionable recommendations to improve inclusivity, flexibility, and interdisciplinary collaboration in teleconsultation.

## Discussion

### Principal Findings

This study was guided by the following research questions: (1) What are the perceived capabilities of nurse-led teleconsultation in supporting poststroke care? (2) What limitations are identified by users? (3) What suggestions do survivors of stroke offer for improving teleconsultation services?

The findings provided clear answers to each research question. First, participants emphasized teleconsultation’s benefits in improving accessibility, emotional comfort, and support for self-management. Second, they reported limitations such as clinical constraints, digital barriers, rigid scheduling, and communication challenges—especially for patients with impairments. Third and last, participants offered concrete suggestions for improvement, such as simplified interfaces, flexible scheduling, patient-initiated access, and closer collaboration between nurses and physicians.

### Comparison to Prior Work

One of the most significant findings is the intersection of digital barriers with patient vulnerability. Although participants in this study had access to devices and internet connectivity, many—particularly older adults—struggled with operating the platform, navigating payment and log-in processes, or resolving technical glitches. These challenges, often perceived as minor by system designers, became substantial obstacles for users with limited digital literacy [23]. Recent reports from the Hong Kong government highlight similar concerns: despite high overall internet penetration, digital competency among older adults remains a key challenge. To address this, the government has launched targeted initiatives under the “Digital 21 Strategy,” while the Digital Economy Development Committee has recommended measures to enhance digital literacy and competency across the community, particularly among older adults and persons with disabilities [24,25]. More importantly, the challenges contributed to feelings of anxiety; dependence; and, in some cases, reluctance to continue using the service. The usability challenges of the HA Go app, Hong Kong’s official digital health platform, exemplify these concerns. While the app was designed to facilitate appointment management, medication records, and teleconsultation, recent user feedback and reports highlight significant usability issues, particularly with real-name registration, in-person activation, and multistep authentication processes, all of which create hurdles for older adults and those with limited digital skills [26]. The Hospital Authority has acknowledged these challenges and is working to improve usability by simplifying workflows and enhancing accessibility, but, as our participants’ experiences suggest, more efforts are needed to ensure that these digital health innovations are truly inclusive. These findings echo broader concerns about the digital divide in health care [27], but they also suggest that teleconsultation, without intentional design for inclusivity, may risk reproducing the very access barriers it aims to overcome. A user-centered approach to technology development—integrating voice-guided interfaces, adopting simplified procedures, and providing community-based training—could help teleconsultation better serve populations considered digitally marginalized, especially older adults managing chronic and complex conditions [28].

Another key issue concerns patient autonomy and the structure of follow-up care. Several participants expressed a desire to initiate consultations based on their own perception of need, rather than waiting for provider-scheduled appointments. This reflects a broader shift in expectations among patients who are increasingly informed, health conscious, and seeking agency in care decisions. While previous studies have highlighted teleconsultation’s potential to empower users [29-31], our findings deepen this understanding by illustrating how institutional rigidity—such as fixed appointment timelines and lack of patient-driven scheduling options—can limit the full potential of teleconsultation. Participants’ calls for more flexible access, including the ability to request sessions during symptom onset or uncertainty, highlight an urgent need to redesign teleconsultation systems to support more dynamic and personalized care pathways.

Crucially, this study also underscores the importance of interdisciplinary collaboration in remote care models. Although nurses were consistently praised for their empathy, attentiveness, and continuity of care, participants recognized that certain decisions—particularly those involving medication adjustments or complex diagnoses—required more immediate access to physicians. Rather than expressing dissatisfaction with nursing care, patients envisioned a more integrated system where nurses and physicians work collaboratively in real time. This vision aligns with emerging models of hybrid care that promote team-based decision-making while respecting the distinctive contributions of each profession [32]. Expanding physician involvement within a nurse-led framework could not only streamline service delivery and reduce delays but also reinforce patient confidence and ensure safe, responsive care for individuals with evolving needs.

Taken together, these findings highlight the dual role of teleconsultation as both a facilitator and potential barrier to equitable, person-centered stroke care. The value of teleconsultation lies not only in its logistical convenience but also in its capacity to strengthen therapeutic relationships, enhance self-management, and reduce the fragmentation of postdischarge support. Nevertheless, realizing these benefits fully will require a more deliberate alignment between system design and patient realities—one that foregrounds usability, flexibility, and interprofessional coordination as essential dimensions of high-quality remote care.

### Strengths and Limitations

A major strength of this study lies in its grounding within a real-world nurse-led teleconsultation program for poststroke care. Conducting focus groups with participants who had recently completed the intervention enhanced the ecological validity of the findings and ensured that the insights reflect actual experiences rather than hypothetical perceptions. The inclusion of diverse voices across sex, educational levels, and age groups further supports the transferability of the results, particularly regarding the varied digital competencies and care needs among survivors of stroke.

Methodologically, the study used rigorous qualitative techniques, including independent coding by two researchers and iterative consensus building to ensure analytical robustness. The use of bilingual researchers in transcription and translation also strengthened the reliability of the data, especially when interpreting nuanced experiences shared in Cantonese.

However, several limitations should be acknowledged. First, the study focuses solely on the perspectives of survivors of stroke and does not include input from caregivers. Given the critical role that caregivers play in facilitating teleconsultation and supporting poststroke recovery, their absence may have limited the comprehensiveness of the findings. Including caregiver perspectives might have revealed additional barriers or strategies related to remote care coordination, digital mediation, and emotional burden. Second, the study also did not include the perspectives of the nurses who conducted the teleconsultations, which could have provided complementary insights into implementation challenges and system-level feasibility. Third, all participants were recruited from a single urban hospital with access to digital infrastructure and relatively high staff-patient continuity. Therefore, the generalizability of the findings to rural or underresourced settings may be limited. Finally, while all participants had access to smartphones and the internet, this inclusion criterion inherently excluded those with more severe socioeconomic or digital disadvantages. As a result, the study may have underrepresented the intensity and prevalence of technological access barriers and usability issues that are likely to affect the populations considered the most vulnerable.

### Future Directions

To enhance the long-term viability and equity of nurse-led teleconsultation, several areas warrant further development. First, technology design should adopt user-centered design principles, with particular attention to older adults and groups considered digitally marginalized. Simplified interfaces, voice-guided navigation, and hotlines for users with low digital literacy could address common barriers, while community-based digital literacy programs would help empower users and should be integrated into discharge planning. These approaches align with Hong Kong’s digital inclusion strategies, which emphasize cross-sectoral collaboration and targeted support [33].

Second, greater flexibility in teleconsultation access is needed. Allowing patients to initiate sessions on demand, rather than relying solely on fixed schedules, could improve autonomy and better accommodate fluctuating health needs, especially for survivors of stroke.

Third, interdisciplinary collaboration should be strengthened. Real-time integration between nurses and physicians within teleconsultation platforms would facilitate timely clinical decisions and enhance continuity of care.

Finally, future research should examine how teleconsultation can be extended to underserved settings and excluded groups, such as those with cognitive impairments, aphasia, or low confidence in digital tools. Studies on hybrid care models and other chronic conditions may also inform scalable, inclusive designs.

### Conclusions

This study deepens understanding of the lived benefits and limitations of nurse-led teleconsultation, highlighting its dual role as both a facilitator and a potential barrier to equitable poststroke care. While most participants appreciated the convenience and reassurance offered by teleconsultation, they also identified critical limitations, including clinical constraints, digital barriers, rigid scheduling, and limited interdisciplinary support.

By centering the voices of survivors of stroke, this study contributes novel patient-centered evidence to the growing telehealth literature and highlights specific system design features—such as flexibility, inclusivity, and nurse-physician collaboration—that can improve the real-world effectiveness of teleconsultation programs. These findings provide actionable insights for policymakers, clinicians, and technology developers to cocreate more equitable, responsive, and user-friendly teleconsultation systems for poststroke care.

## Appendix

Multimedia Appendix 1 Interview questions.

Multimedia Appendix 2 COREQ checklist.

## Data Availability

The datasets generated and analyzed during this study are not publicly available due to terms of consent for study participation but are available from the corresponding author on reasonable request.
