# Supplementary material for: Navigating the Boundaries of Teleconsultation—Capabilities, Limitations, and Pathways for Improvement: Qualitative Study of the Experiences of Patients With Stroke
Source: J Med Internet Res. 2025 Sep 18;27:e75841. doi: 10.2196/75841 (PMC12491883; doi:10.2196/75841)
Supplement: Multimedia Appendix 1 [file jmir_v27i1e75841_app1.docx]

Multimedia Appendix 1: Interview Questions

Part 1: Background experiences

- Could you please tell me about the procedure or process when receiving teleconsultations?
- Have you had any former experience with teleconsultations before this project?
- It is hard for you to operate teleconsultations? Have you encountered any barriers or shortcomings while engaging in the teleconsultation program? Have these difficulties/barriers been alleviated or resolved finally? If not, how can we help you to address them?

Part 2: Perception

- What do you think of teleconsultations? Do you find it suitable for you as a stroke patient?
- How do the teleconsultations take care of your needs?
- What do you like or dislike about the intervention program?
- In comparison to traditional face-to-face consultations, can you identify any advantages and shortcomings?
- Which consultation format do you prefer? Teleconsultations or face-to-face consultations? Why?

Part 3: Suggestion

- In your opinion, what changes or improvements would you suggest for the teleconsultations program to better meet your needs and benefit you the most?

Part 4: Feedback

- Would you be willing to join similar teleconsultation programs in the future? Why?
- Would you recommend the teleconsultations program for stroke patients to be continued and introduced to other stroke patients for nurse clinic for follow-up? Why?
- What do you think teleconsultations can be done and not done when compared to face-to-face consultation?
- What are your needs that are not met by teleconsultations?

Part 5: Conclusion

- To help our study on the teleconsultations, we would like to have your brief summary on the following questions:

1. Your comments on THIS teleconsultations program;
2. Your comments on implementing teleconsultations in the population of stroke patients, is this population suitable for teleconsultations? If not, what kind of population may be more suitable?
3. Your comments on teleconsultations in total.
