# Supplementary material for: Navigating the Boundaries of Teleconsultation—Capabilities, Limitations, and Pathways for Improvement: Qualitative Study of the Experiences of Patients With Stroke
Source: J Med Internet Res. 2025 Sep 18;27:e75841. doi: 10.2196/75841 (PMC12491883; doi:10.2196/75841)
Supplement: Multimedia Appendix 2 [file jmir_v27i1e75841_app2.docx]

Multimedia Appendix 2. The Consolidated Criteria for Reporting Qualitative Studies (COREQ): 32-item checklist

| No | Item | Guide questions/ description | Notes |
| --- | --- | --- | --- |
| **Domain 1: Research team and reflexivity** | | |  |
| Personal Characteristics | | |  |
| 1. | Interviewer/facilitator | Which author/s conducted the interview or focus group? | All interviews were conducted by the first author and the second author of this research. (P. 4) |
| 2. | Credentials | What were the researcher's credentials? *E.g. PhD, MD* | The first author holds a PhD while the second author holds a MPhil at the time of study. (Cover page) |
| 3. | Occupation | What was their occupation at the time of the study? | The first author was an associate professor and the second author was a research associate. (Cover page) |
| 4. | Gender | Was the researcher male or female? | The first author was a male and the second author was a female. (N/A) |
| 5. | Experience and training | What experience or training did the researcher have? | The first author and the second author had experience with qualitative methods including facilitating focus groups and conducting interviews. (N/A) |
| Relationship with participants | | | |
| 6. | Relationship established | Was a relationship established prior to study commencement? | The participants participated in a clinical trial prior to the qualitative interview. (N/A) |
| 7. | Participant knowledge of the interviewer | What did the participants know about the researcher? *e.g. personal goals, reasons for doing the research* | To set time for interviews, some participants received call contacts. Research subject, objectives, and identity of author were given in contacts. Additionally, author 1–6 were the main researchers in the clinical trial. (P.4) |
| 8. | Interviewer characteristics | What characteristics were reported about the interviewer/facilitator? *e.g. Bias, assumptions, reasons and interests in the research topic* | The research team had an interest in the participants’ experiences of nurse-led teleconsultation services and capabilities and limitations from the patients’ perspectives. (P.4) |
| **Domain 2: Study design** | | | |
| Theoretical framework | | | |
| 9. | Methodological orientation and Theory | What methodological orientation was stated to underpin the study? *e.g. grounded theory, discourse analysis, ethnography, phenomenology, content analysis* | Content analysis was used to analyze the qualitative data. (P.3) |
| Participant selection | | | |
| 10. | Sampling | How were participants selected? *e.g. purposive, convenience, consecutive, snowball* | All of the participants were from the participants of a prior clinical trial, who had used the telerehabilitation service studied in this paper. (P.3) |
| 11. | Method of approach | How were participants approached? *e.g. face-to-face, telephone, mail, email* | The participants were contacted by telephone. (P.3) |
| 12. | Sample size | How many participants were in the study? | There were 21 participants in this study. (P.4) |
| 13. | Non-participation | How many people refused to participate or dropped out? Reasons? | None of them refused to participate or dropped out. (P.4) |
| Setting | | | |
| 14. | Setting of data collection | Where was the data collected? *e.g. home, clinic, workplace* | The data was collected via online Zoom platform. (P.3) |
| 15. | Presence of non-participants | Was anyone else present besides the participants and researchers? | The participants attended the meeting by themselves. (N/A) |
| 16. | Description of sample | What are the important characteristics of the sample? *e.g. demographic data, date* | The demographic information of the sample was provided in Table 1. (P.4-5) |
| Data collection | | | |
| 17. | Interview guide | Were questions, prompts, guides provided by the authors? Was it pilot tested? | The questions, prompts, guides were provided by the authors. They were pilot tested with  non-participants. (Appendix 1) |
| 18. | Repeat interviews | Were repeat interviews carried out? If yes, how many? | No. (N/A) |
| 19. | Audio/visual recording | Did the research use audio or visual recording to collect the data? | The research used audio and visual recordings.(N/A) |
| 20. | Field notes | Were field notes made during and/or after the interview or focus group? | The researcher took field notes during the interview. (P.3) |
| 21. | Duration | What was the duration of the interviews or focus group? | Each interview took 1-2 hours. (P.3) |
| 22. | Data saturation | Was data saturation discussed? | We coded the data until it reached saturation. (P.3) |
| 23. | Transcripts returned | Were transcripts returned to participants for comment and/or correction? | No. (N/A) |
| **Domain 3: analysis and findings** | | | |
| Data analysis | | | |
| 24. | Number of data coders | How many data coders coded the data? | Two coders coded the data: the first author and the second author. (P.3) |
| 25. | Description of the coding tree | Did authors provide a description of the coding tree? | No. (N/A) |
| 26. | Derivation of themes | Were themes identified in advance or derived from the data? | The themes were derived from the data. (P.5-9) |
| 27. | Software | What software, if applicable, was used to manage the data? | NVivo 12 was used to manage the data. (P.3) |
| 28. | Participant checking | Did participants provide feedback on the findings? | No. (N/A) |
| Reporting | | | |
| 29. | Quotations presented | Were participant quotations presented to illustrate the themes / findings? Was each quotation identified? *e.g. participant number* | Yes. (P.4-5) |
| 30. | Data and findings consistent | Was there consistency between the data presented and the findings? | Yes. (P.5) |
| 31. | Clarity of major themes | Were major themes clearly presented in the findings? | Yes. (P.5-9) |
| 32. | Clarity of minor themes | Is there a description of diverse cases or discussion of minor themes? | Yes. (P.5-9) |
